# Hemolytic Activity in Relation to the Photosynthetic System in *Chattonella marina* and *Chattonella ovata*

**DOI:** 10.3390/md19060336

**Published:** 2021-06-12

**Authors:** Ni Wu, Mengmeng Tong, Siyu Gou, Weiji Zeng, Zhuoyun Xu, Tianjiu Jiang

**Affiliations:** 1 Key Laboratory of Aquatic Eutrophication and Control of Harmful Algal Blooms of Guangdong Higher Education Institute, Research Center of Hydrobiology, Jinan University, Guangzhou 510632, China; wuniapple@163.com (N.W.); gsy@stu2018.jnu.edu.cn (S.G.); zwjjn@stu2018.jnu.edu.cn (W.Z.); 2 South China Sea Institute of Planning and Environmental Research, State Oceanic Administration, Guangzhou 510300, China; 3 Ocean College, Zhejiang University, Zhoushan 316021, China; xu_zy@zju.edu.cn

**Keywords:** *Chattonella marina*, *Chattonella ovata*, hemolytic activity, photosystem II, hydrogen peroxide, chlorophyll *c2*

## Abstract

*Chattonella* species, *C. marina* and *C. ovata*, are harmful raphidophycean flagellates known to have hemolytic effects on many marine organisms and resulting in massive ecological damage worldwide. However, knowledge of the toxigenic mechanism of these ichthyotoxic flagellates is still limited. Light was reported to be responsible for the hemolytic activity (HA) of *Chattonella* species. Therefore, the response of photoprotective, photosynthetic accessory pigments, the photosystem II (PSII) electron transport chain, as well as HA were investigated in non-axenic *C. marina* and *C. ovata* cultures under variable environmental conditions (light, iron and addition of photosynthetic inhibitors). HA and hydrogen peroxide (H_2_O_2_) were quantified using erythrocytes and pHPA assay. Results confirmed that% HA of *Chattonella* was initiated by light, but was not always elicited during cell division. Exponential growth of *C. marina* and *C. ovata* under the light over 100 µmol m^−2^ s^−1^ or iron-sufficient conditions elicited high hemolytic activity. Inhibitors of PSII reduced the HA of *C. marina*, but had no effect on *C. ovata*. The toxicological response indicated that HA in *Chattonella* was not associated with the photoprotective system, i.e., xanthophyll cycle and regulation of reactive oxygen species, nor the PSII electron transport chain, but most likely occurred during energy transport through the light-harvesting antenna pigments. A positive, highly significant relationship between HA and chlorophyll (chl) biosynthesis pigments, especially chl *c2* and chl *a*, in both species, indicated that hemolytic toxin may be generated during electron/energy transfer through the chl *c2* biosynthesis pathway.

## 1. Introduction

The raphidophycean flagellates *Chattonella marina* and *C. ovata* [1,2,3,4,5], and other flagellates such as *Heterosigma akashiwo* [6], *Heterocapsa circularisquama* [7], *Phaeocystis globosa* [8], *Amphidinium carterae* [9,10], *Prymnesium parvum*, and *Chrysochromulina polylepis* [11] have been reported as the causative species of massive, fish-killing algal blooms worldwide. The major ichthyotoxic effects of these flagellates were identified as: (1) producing reactive oxygen species (ROS) [5,12,13,14,15], (2) clogging of the gills [4,16,17,18], (3) causing neurotoxin-induced cardiac disorders [19], and (4) producing hemolytic toxins [20,21,22,23] that result in necrosis of the gills. All these effects function either separately or synergistically, resulting in gill tissue injury or direct/indirect toxicity to the fish.

The toxigenic and toxicological mechanisms of action of these fish-killing species; however, remain largely unclear due to the toxins’ instability, multiple structures and/or synergistic or antagonistic effects. Hemolytic compounds extracted from *C. marina* were identified as polyunsaturated fatty acids [13,18], or lipids and glycolipids [24], or chlorophyll (chl) *c* derivatives [20]. Those extracted from *Heterocapsa circularisquama* were characterized as a porphyrin derivative with a chemical structure similar to a pyropheophorbide *a* methyl ester [7]. Furthermore, some phycotoxins are light-dependent and associated with photosynthesis, i.e., okadaic acid (OA) was located in the chloroplasts of *Prorocentrum lima* cells [25]; the N-sulfocarbamoyl toxin C2, a paralytic shellfish toxin, and hemolytic compounds were associated with the production of chl *a* in the dinoflagellate *Alexandrium tamarense* [26], and chl *c2* in kelp, *Eisenia bicyclis* [27], respectively. Monogalactosyldiacylglycerins (MGDG) and digalactosyl diacylglycerins (DGDG), the major lipid constituents of the photosynthetic membrane of *Fucus evanescens* [28], *Karenia mikimotoi* (formerly *Gymnodinium mikimotoi*) and *Gymnodinium* sp. [29] could also induce hemolytic activity. The above evidence thus indicates that the production of these phycotoxins may occur during photosystem of eukaryotic algae.

The role of reactive oxygen species (ROS) in phycotoxin production, especially that of ichthyotoxins, remains poorly understood. Eukaryotic phytoplankton commonly produce ROS under optimal environmental conditions [30]; for example, *C. marina*, *C. antiqua* and *Heterosigma akashiwo* produced ROS during the exponential growth phase and this production remained constant during the stationary phase [31,32,33]. Internal or external stressors, associated with biological interactions or environmental factors, could also lead to an increase in ROS production [34]. The antioxidative defense system, such as the glutathione-ascorbate (GSH-ASA) and xanthophyll cycles, associated with photoprotection, are initiated by ROS and allow energy dissipation by non-photochemical chlorophyll fluorescence quenching (NPQ) [35,36,37]. The production of ROS is not directly cytotoxic but is considered to be indirectly associated with toxic effects, e.g., by stimulating the production of lipid peroxidation products [30].

The ichthyotoxic effects were reported to be species or strain-specific in *Chattonella* [38], *Phaeocystis* [39], and other flagellates [40]; however, they are not solely related to the hemolytic activity or synergistic effects of HA and ROS [13,41,42,43,44]. The role of predators [45], prey, or the presence of bacteria, including nutrient competition [46,47], nutrient supply, algae killer or allelopathic inducers [48,49,50,51], may also act as the key driver to the bloom dynamics or toxicological mechanisms of those toxic flagellates, resulting in great deferring response of growth and ichthyotoxicity.

**Figure 1:**
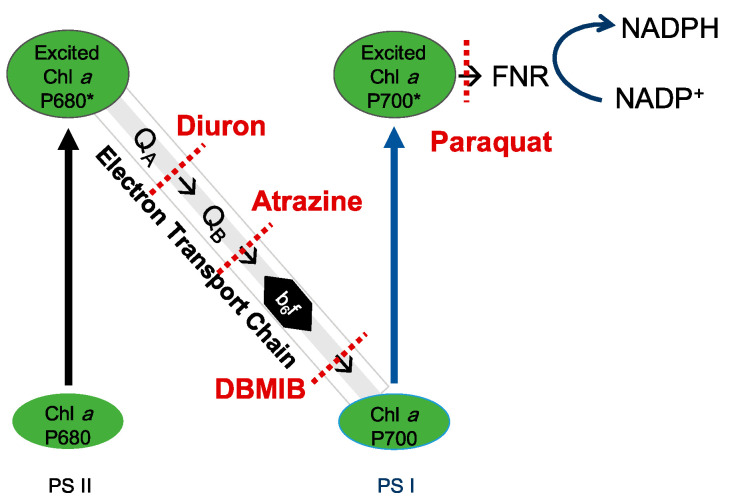
Schematic of the photosynthetic system in *Chattonella* and potential blocking spots of four photosynthetic electron transport inhibitors. NADP^+^: nicotinamide adenine dinucleotide phosphate; NADPH: nicotinamide adenine dinucleotide phosphate H; NPQ: light-induced non-photochemical fluorescence quenching; b_6_f: cytochrome b_6_f complex; FNR: ferredoxin-NADP^+^ oxidoreductase; PSI and PSII: photosystems I and II. * indicates the high energy level of P680 or P700.

Therefore, in the present study the light-induced photosynthetic system, including the accessory pigments, the relative electron transfer rate (rETR), photosynthetic efficiency (F_v_/F_m_), quantum yield of photosystem II (PSII Yield), hydrogen peroxide (H_2_O_2_) production (an indicator of ROS) and the stress-induced xanthophyll cycle, together with the hemolytic activity of *C. marina* and *C. ovata* were investigated under variable environmental conditions. To simplify the photosynthesis process, the photosynthetic system is illustrated by the electron/energy transport pathway through the Z-Scheme of *Chattonella* (Figure 1), the light-harvesting antenna pigment, the electron transport chain, and photoprotective or antioxidative system. The overall aim of this study is thus to identify which photosynthetic process(es) is/are associated with hemolytic activity in *Chattonella*.

## 2. Results

### 2.1. Effects of Light

#### 2.1.1. Growth Response

**Figure 2:**
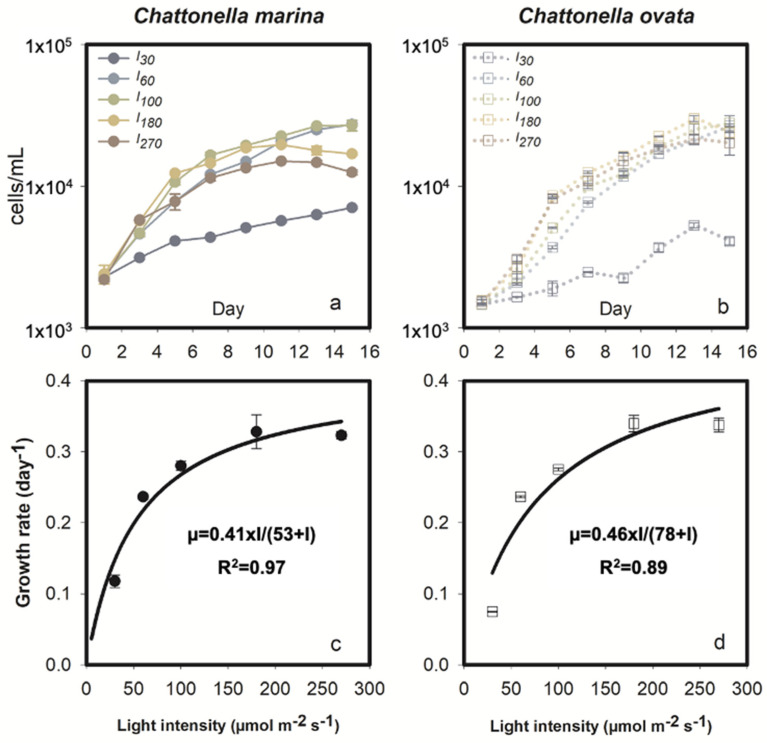
Growth response of *Chattonella marina* (**a**,**c**) and *C. ovata* (**b**,**d**) under different light intensities (I). Values represent the mean ± standard deviation. Fitted growth curves and the coefficient of determination (R^2^) are also shown in (**c**,**d**).

As expected, growth of the phototrophic *C. marina* was significantly affected by light intensity (Figure 2a,c). *Chattonella marina* grew rapidly during the early exponential phase, i.e., during the first 3 to 7 days experiencing 0.3 to 2 divisions, then continued to grow at a lower rate for 6 days, reaching a maximum concentration of 27,000 cells mL^−1^ under light intensities of *I_60_* and *I_100_*, followed by ~15,000 cells mL^−1^ at *I_180_* and *I_270_*, and ~7000 cells mL^−1^ under low light, *I_30_* (Figure 2a). Growth rate (*µ*) values for this species were 0.12, 0.24, 0.28, 0.33 and 0.32 at the five irradiance levels *I_30_*, *I_60_*, *I_100_*, *I_180_* and *I_270_*, respectively. Based on the Michaelis Menten (M-M) model, the maximum *µ* was 0.41 day^−1^ with a half saturation light intensity of 53 µmol m^−2^ s^−1^ (Figure 2c).

Growth of *C. ovata* increased with increasing light intensity ranging from 30 to 180 µmol m^−2^ s^−^^1^, with a maximum growth rate of 0.07 to 0.34 day^−1^ (Figure 2b,d). However, *I_270_* stressed the cells of *C. ovata*, as evidenced by comparable growth rates (*p* > 0.05) at *I_270_* and *I_180_* during early exponential growth (Figure 2b,d). *Chattonella ovata* grew at a slower rate during the mid-exponential phase, i.e., between 5 and 13 days, reaching an extremely high concentration of 25,000 cells mL^−1^ at *I_60_* to *I_180_* (Figure 2b). Growth dynamics of *C. ovata* in response to light is shown in Figure 2d. The maximum *µ* of *C. ovata* was 0.46 day^−1^, greater than that of *C. marina*, with a half saturation constant of 78 µmol m^−2^ s^−1^ (Figure 2d).

#### 2.1.2. Photosystem II Energy Fluxes and Photopigments

**Figure 3:**
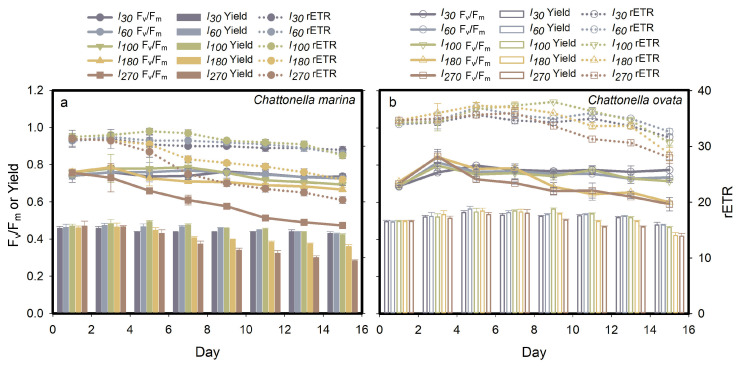
Effect of light intensity (I) on the photosynthetic parameters of *Chattonella marina* (**a**) and *C. ovata* (**b**): photosynthetic efficiency (F_v_/F_m_), photosystem quantum yield (Yield) and relative electron transfer rate (rETR). Values indicate the mean ± standard deviation.

The pattern of photosystem II energy fluxes, F_v_/F_m_, Yield, and rETR, of *C*. *marina* and *C. ovata* are shown in Figure 3. In general, the photosynthetic activity (F_v_/F_m_) of exponentially growing cells of the two species was constant and high under optimal light conditions, i.e., 30–80 µmol m^−2^ s^−1^ for the former (Figure 3a) and 30–100 µmol m^−2^ s^−1^ for the latter (Figure 3b), attaining a mean ± standard deviation (SD) of 0.74 ± 0.03 and 0.75 ± 0.03, respectively. The highest light intensity tested, *I_270_*_,_ in *C. marina* and both *I_180_* and *I_270_* in *C. ovata*, significantly (*p* < 0.05) affected the photosynthetic efficiency as cells became senescent. The quantum yield of PSII and rETR of *Chattonella* were also inhibited under high light stress (Figure 3), resulting in a significant down-regulated trend with growth stage progression.

**Figure 4:**
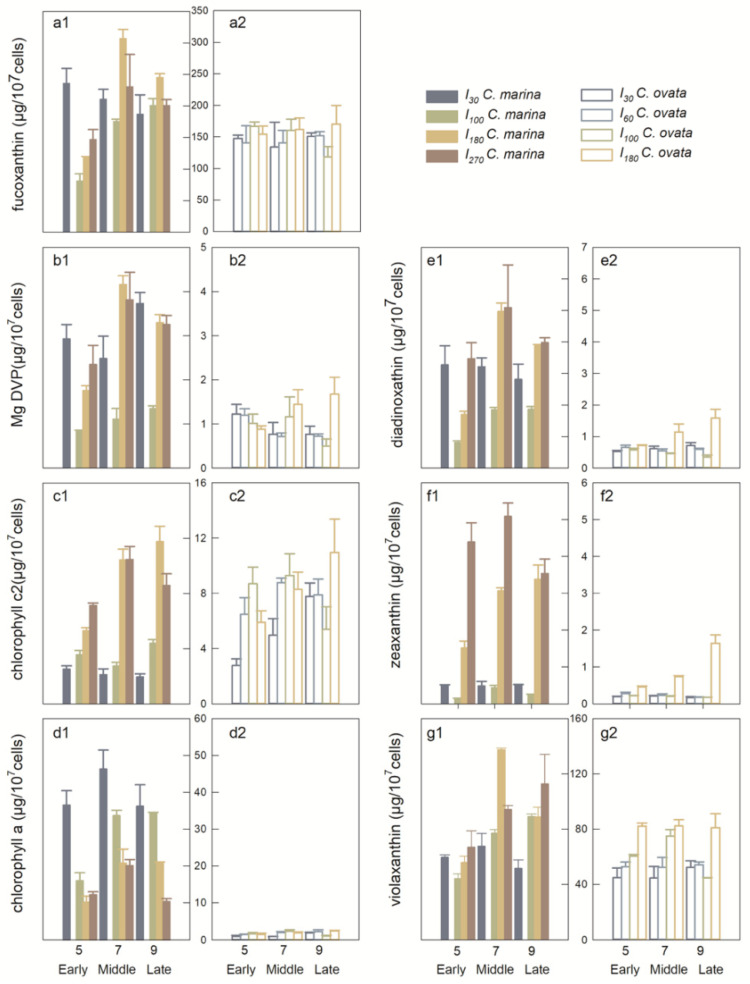
Concentrations (mean ± standard deviation) of fucoxanthin (**a**), Mg-2,4-divinylpheoporphyrin (Mg DVP) (**b**), chlorophyll *c2* (**c**), chlorophyll *a* (**d**), diadinoxathin (**e**), zeaxanthin (**f**), violaxanthin (**g**), per 10^7^ cells of *Chattonella marina* (1) and *C. ovata* (2) under different light intensities on days 5, 7 and 9 (corresponding to early, mid- and late exponential growth phases of two *Chattonella* species).

Seven out of seventeen photopigments were detected in *C. marina* and *C. ovata* samples by HPLC, including fuco, viola, diadino, Mg DVP, zea, chl *a* and chl *c2*. Pigment concentrations varied greatly with light intensity and growth phase (Figure 4). Fucoxanthin and viola are the dominant pigments of *C. marina* and *C. ovata*, with an average prevalence of 62% to 25% and 68% to 27%, respectively. Diadinoxanthin and zea comprised <1% of total pigments, but varied markedly between *C. ovata* (Figure 4(e2,f2)) and *C. marina* (Figure 4(e1,f1)). It is noteworthy that the cellular chl *a* content was 10× less in *C. ovata*, than in *C. marina* (Figure 4(d1,d2)).

Photopigments were further grouped in the present study to better understand energy transport during photosynthesis. Photoprotective pigments (PPPs), i.e., those involved in the xanthophyll cycle, namely viola, diadino, zea, and the light-harvesting antenna with chlorophylls *c* (LHC*cs*), i.e., Mg DVP, chl *c2* and chl *a*. Violaxanthin contributed the major portion, over 95% of PPPs; LHC*cs* averaged ~11 ± 3% and 4 ± 1% of total pigments of *C. marina* and *C. ovata*, respectively. Chlorophyll *a* was the dominant LHC pigment in *C. marina*, accounting for 71% of the total, whereas Chl *c2* was dominant in *C. ovata*, comprising up to 67% of total LHCs.

#### 2.1.3. Hemolytic Activity and H_2_O_2_ Production

**Figure 5:**
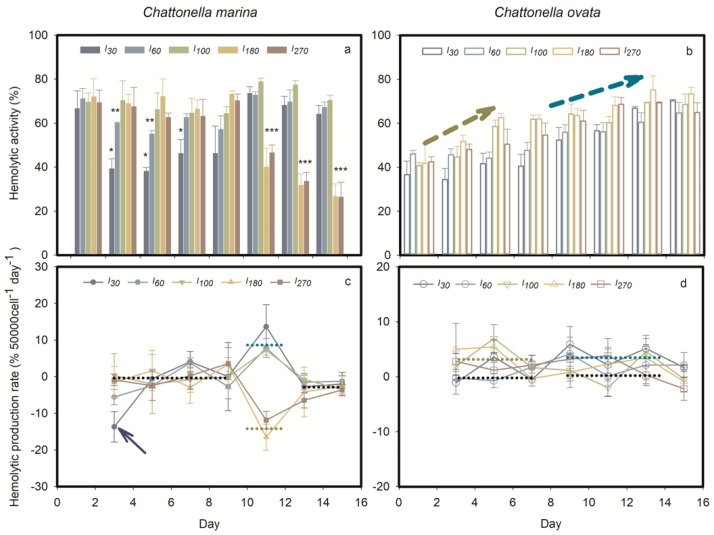
Time course of mean (± standard deviation) percent hemolytic activity (**a**,**b**) and toxin production rate (**c**,**d**) of *Chattonella marina* and *C. ovata* under different light intensities (I). *, ** and *** indicate the significance of the differences, *p* < 0.05, *p* < 0.01 and *p* < 0.001, respectively. The yellow arrows indicate a significant difference with an average value among five light treatments over 0 to 4 days. The dark blue arrows indicate significant differences among average values of five light treatments over 8 to 14 days.

Light significantly affected the hemolytic activity of *C. marina* and *C. ovata* (Figure 5). The maximum toxin quota was found during exponential growth of *C. marina* under high light (*I_100_*, *I_180_* and *I_270_*) and the stationary phase under low light condition (*I_30_* and *I_60_*). The latter limited *C. marina* growth and hemolytic activity during the exponential phase, attaining 38.6% and 57% (* and ** in Figure 5a), at the two low light levels, respectively. High light, i.e., *I_100_*, *I_180_* and *I_270_*, supported *C. marina* growth, and resulted in accumulation of hemolytic toxin during the exponential growth phase, but its production was reduced significantly when cells became senescent (*** in Figure 5a). Production rate of hemolytic compounds was calculated from these data, showing that the toxin was not produced during exponential growth of *C. marina* except at extremely low light levels (see arrow in Figure 5c). Toxin was produced during the early stationary stage under low light but declined in the late stationary stage in *C. marina*. When cells became senescent, hemolytic toxins were no longer produced in any of the light treatments (Figure 5c).

In contrast to the toxinological pattern in *C. marina*, hemolytic compound was continuously produced in *C. ovata* at all light intensities tested (Figure 5b). Generally, growth of *C. ovata* was divided into three stages: early exponential (day 0–5), mid-late exponential (day 5–13) and early stationary (day 13–15). During early exponential growth *C. ovata* showed significant toxin production at high light intensities (marked by the dark yellow arrow in Figure 5b and dotted yellow line in Figure 5d), i.e., *I_100_*_, *180*, *270*_. In contrast, HA in *C. ovata* was significant during mid-late exponential growth under low light (dark blue arrow in Figure 5b and dotted blue line in Figure 5d); *C. ovata* stopped producing the hemolytic toxin during the stationary phase (*p* > 0.05). 

**Figure 6:**
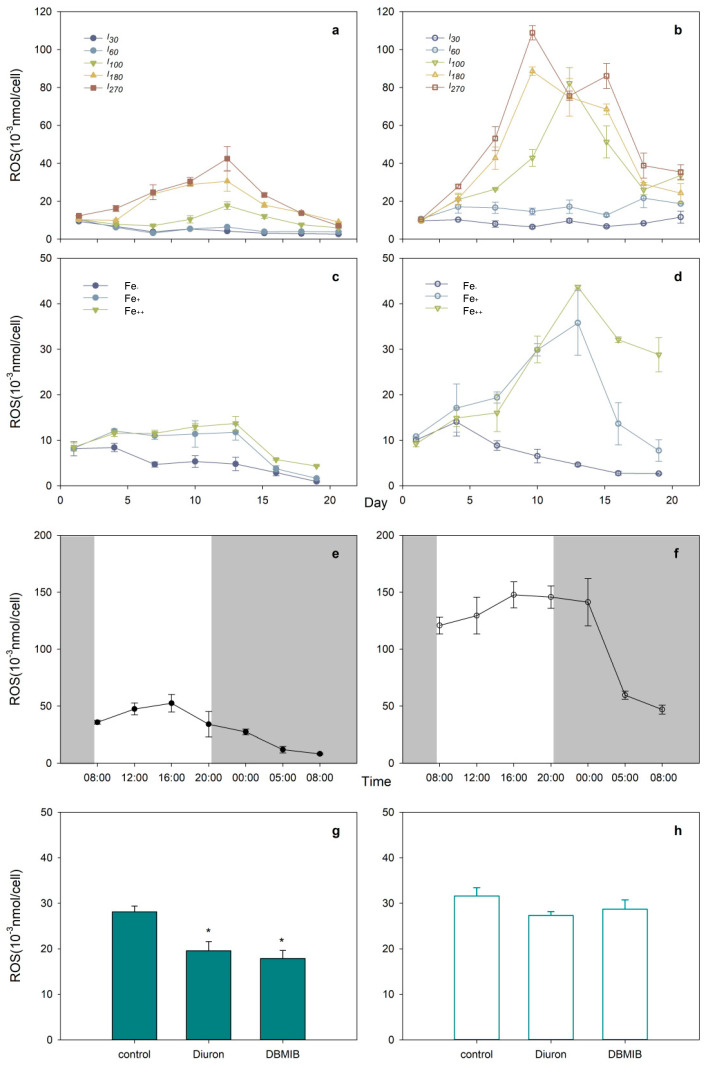
Time course of mean (±standard deviation) cellular molar concentrations of hydrogen peroxide (H_2_O_2_), a representative reactive oxygen species (ROS), in *Chattonella marina* (**a**,**c**,**e**,**g**) and *C. ovata* (**b**,**d**,**f**,**h**) under different light intensities, I (**a**,**b**), iron, Fe additions (**c**,**d**), light/dark cycle (light indicated by grey shading) (**e**,**f**) and three photosynthetic electron transport inhibitors (**g**,**h**), where * indicates significant differences at *p* < 0.05.

Oxidative activity, as measured by cellular H_2_O_2_ production of *C. marina* and *C. ovata,* is shown in Figure 6. Higher ROS concentrations were determined during exponentially growing *C. marina* and *C. ovata* cells under higher light intensity (Figure 6a,b). The two species, however, exhibited a differential ROS response, such that the variation in ROS concentrations in *C. ovata* (maximum of 108 pmol cell^−1^) was significantly greater than that in *C. marina* (41 pmol cell^−1^ maximum).

### 2.2. Light:Dark Cycle Effects

**Figure 7:**
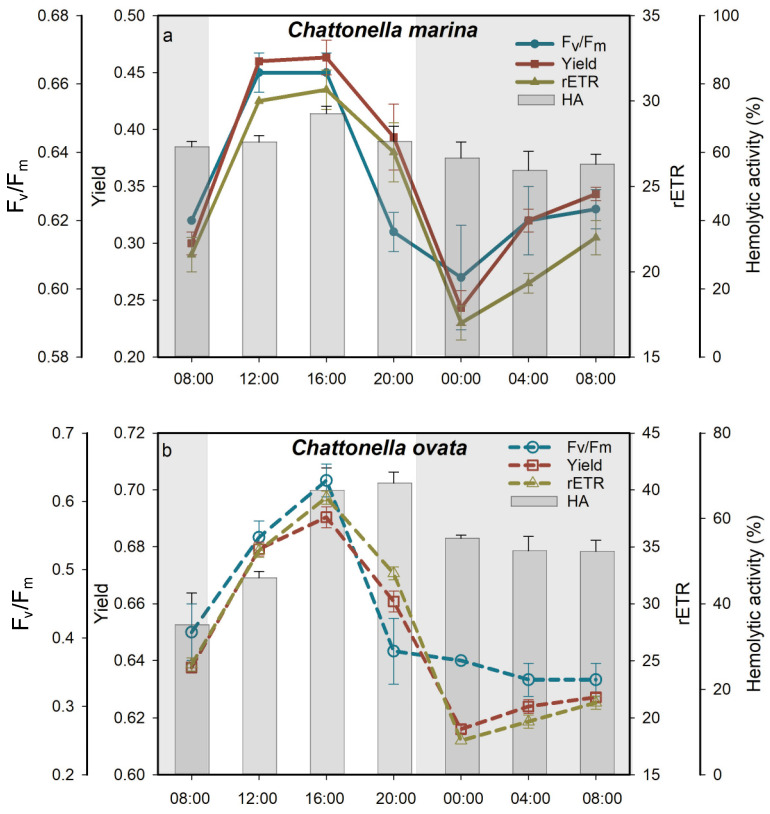
Variation of photosynthetic efficiency, F_v_/F_m_, quantum yield, Yield, relative electron transfer rate, rETR and percent hemolytic activity, HA, of *Chattonella marina* (**a**) and *C. ovata* (**b**) over 24 h, 12:12 light/dark cycle. Grey shading represents the dark period; the light period started at 09:00 a.m.

The response of F_v_/F_m_, Yield and rETR over a 24 h 12:12 light:dark cycle exhibited a classical sigmoidal shape, increasing during the light cycle and decreasing during the dark cycle in both species (Figure 7). Similarly, the hemolytic activity of *C*. *marina* (Figure 7a) and *C. ovata* (Figure 7b) increased with increasing light exposure and reached a maximum after 7 h of the photosynthetic process (*p* < 0.05), then decreased (significantly in *C. ovata*, but not in *C. marina*) during the following 11 h (Figure 7). The lowest hemolytic activity was detected after 7 h of the dark period. The average (± SD) HA during the light period reached 65.8 ± 4.8% and 58.7 ± 8.4% in *C. marina* and *C. ovata*, respectively, values which were significantly (*p* < 0.05) greater than those observed in the dark (57.8 ± 3.0% and 50.0 ± 13.1%, respectively). 

*Chattonella marina* and *C. ovata* also displayed a sigmoidal pattern of H_2_O_2_ production (Figure 6e,f). Cells were capable of generating more ROS during the day than at night, and ROS production by *C. ovata* was 3.6× higher on average than that by *C. marina*.

### 2.3. Effects of Iron

#### 2.3.1. Growth Response

**Figure 8:**
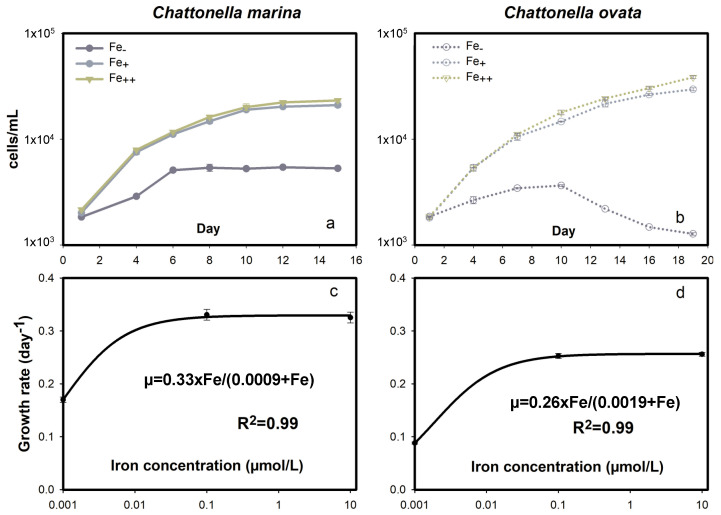
Growth response of *Chattonella marina* (**a**,**c**) and *C. ovata* (**b**,**d**) exposed to different iron (Fe) concentrations. Fitted growth curves and the coefficient of determination (R^2^) are also shown.

Free Fe or low Fe_-_ conditions inhibited *C. marina* and *C. ovata* growth rates, which dropped to minima of 0.17 and 0.09 day^−1^ at maximum cell concentrations of 5100 and 3500 cells mL^−1^, respectively (Figure 8a,b). In contrast, significant growth was observed with iron (Fe_+_ and Fe_++_) additions. The simulated M-M model showed that the maximum *µ* was 0.33 and 0.26 day^−1^ with a predicted (not measured) half saturation Fe concentration of 0.9 and 1.9 nmol L^−1^ for *C. marina* and *C. ovata*, respectively (Figure 8c,d). The relatively low maximum growth rate and high half saturation Fe concentration of *C. ovata* compared to *C. marina* were indicative of a greater Fe requirement and K-selective uptake characteristics of *C. ovata*. 

#### 2.3.2. Photosystem II Energy Fluxes

**Figure 9:**
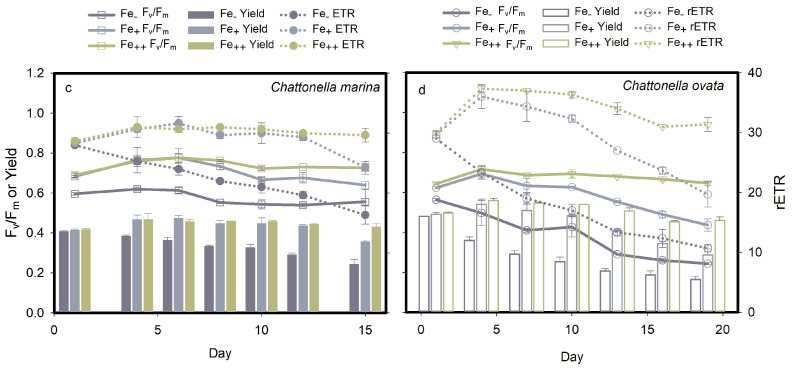
Effect of iron (Fe) on photosynthetic parameters (mean ± standard deviation) F_v_/F_m_, Yield and rETR of *Chattonella marina* (**a**) and *C. ovata* (**b**): photosynthetic efficiency, F_v_/F_m_, quantum yield, Yield, and relative electron transfer rate, rETR.

Iron played a key role in determining the photosynthetic activity of *Chattonella*, as shown by significant down-regulation (*p* < 0.05) of F_v_/F_m_, Yield and rETR under Fe-deficient conditions (Figure 9a,b). High iron concentrations helped to stimulate a greater photosynthetic activity in both *C. marina* and *C. ovata*, with the highest F_v_/F_m_ values of 0.78 ± 0.02 and 0.72 ± 0.02, respectively.

#### 2.3.3. Hemolytic Activity and H_2_O_2_ Production

**Figure 10:**
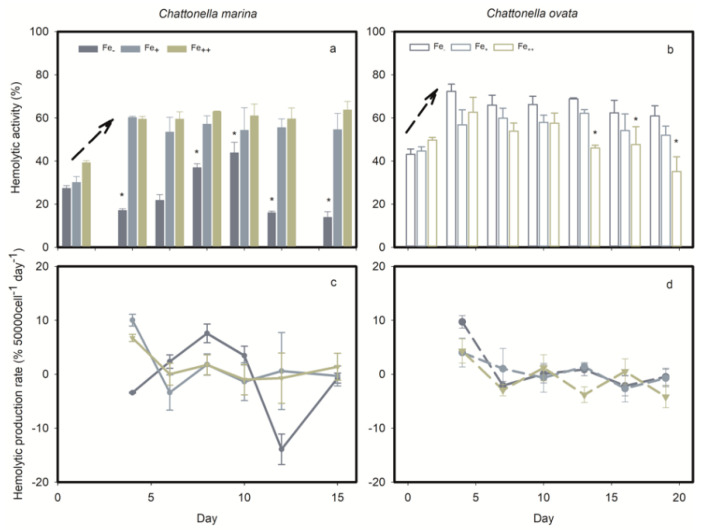
Percent hemolytic activity (**a**,**b**) and toxin production rate (**c**,**d**) of *Chattonella marina* and *C. ovata* exposed to different iron (Fe) treatments. * indicates significant differences at *p* < 0.05. Dark arrows indicate a significant increase over 0 to 4 days, except for Fe treatments in *C. marina*.

The HA response of *C. marina* and *C. ovata* under iron stress are shown in Figure 10. Significant HA by both *Chattonella* species during the early exponential phase (day 0–4) occurred under all iron conditions tested (arrows in Figure 10a,b). A differential response was observed, however, in the Fe treatment of *C. marina*, where hemolytic activity was low during the exponential growth phase, then increased significantly (*p* < 0.05) until the early stationary phase (day 10). During cell senescence, hemolytic toxin was released from *C. marina* cells under low light conditions (* in Figure 10a) and from *C. ovata* under high light conditions (* in Figure 10b).

Iron stress, neither Fe-deplete nor Fe-sufficient treatments, led to the generation of significant H_2_O_2_ concentrations in *C. marina*; however, as observed in light treatments, 3–4 × higher H_2_O_2_ concentrations were produced under Fe-sufficient conditions in *C. ovata* (Figure 6c,d). Maximum H_2_O_2_ concentrations were detected on day 13, i.e., around late exponential growth of *C. marina* and *C. ovata*.

### 2.4. Effects of Photosynthetic Electron Transport Inhibitors

**Figure 11:**
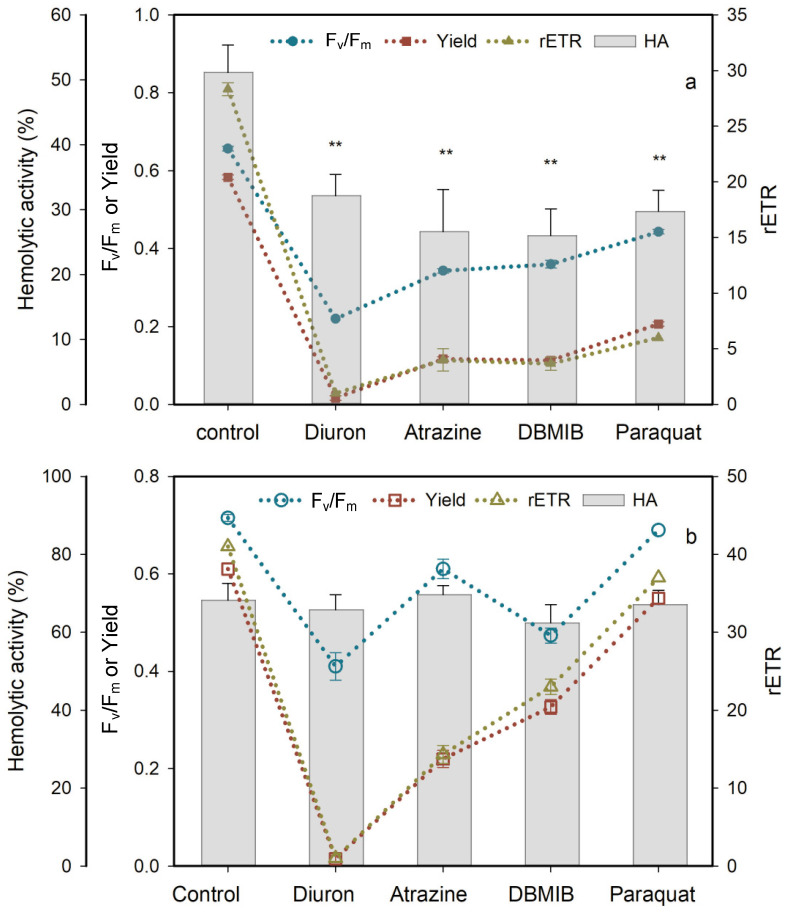
Effect of four photosynthesis blockers on percent hemolytic toxicity, HA, photosynthetic efficiency F_v_/F_m_, quantum yield, Yield, and elative electron transfer rate, rETR (mean ± standard deviation) of *Chattonella marina* (**a**) and *C. ovata* (**b**) after one hour of exposure, relative to the control. ** indicates a significant difference from the control (*p* < 0.01).

Four PSII inhibitors, diuron, atrazine, DBMIB and paraquat, significantly blocked the photosynthetic activity and HA of *C*. *marina* within 1 h of exposure (Figure 11a). Photosynthetic efficiency of *C*. *marina* decreased from a healthy condition (0.65) to stress levels of 0.2 (diuron), 0.3 (atrazine and DBMIB) and 0.4 (paraquat). In contrast, the effects of the four PSII inhibitors on F_v_/F_m_ of *C. ovata* was significant but less pronounced, from 0.72 to 0.41 (*p* < 0.5), 0.61 (*p* < 0.5), 0.47 (*p* < 0.5) and 0.69 (*p* > 0.5), respectively (Figure 11b). Yield and rETR of *C. marina* and *C. ovata* were fully blocked under the stress of diuron exposure, followed by atrazine, DBMIB and paraquat (Figure 11). It is especially noteworthy that the hemolytic activity of *C. ovata* was not affected by exposure to the four PSII inhibitors (Figure 11b).

## 3. Discussion

Exogenous stress in *Chattonella marina* and *C. ovata* cells, stimulated the expression of the photosynthetic system, including photosystem I, electron/energy transport chain and photosystem II, resulting in a metabolic imbalance. Results of the present study confirm the hypothesis that hemolytic compounds are generated during photosynthesis and further identify the photosynthesis process that may be associated with hemolytic activity in *Chattonella*.

### 3.1. Ecological Significance of the Growth and Hemolytic Activity Response

Irradiance and iron are essential for most phytoplankton, especially phototrophic phytoplankters. Saturation light of *C. marina* was reported at 30~110 µmol m^−2^ s^−1^ under the suitable temperature (20~30 °C), salinity (20–35 psu) and nutritional condition, with the growth rate ranging from 0.3 to 1.4 d^−1^ [52,53]. The maximum growth rate of *C. marina*, our Hong Kong isolate, reached 0.41 d^−1^ (Figure 4c), located at the lower range of all the reported *C. marina* strains. Similarly, low growth (max. 0.46 d^−1^, Figure 4d) was observed at the strain of *C. ovata*, compared to the isolates from Japan (0.8~1.4 d^−1^) under the similar condition, indicating the strain genetic difference and/or potential biological stress, such as co-existing bacteria [52,53]. The non-axenic *C. marina* and *C. ovata* culture in the present study were established at 2002 and 2003 [54,55], with no significant changes on growth rate and HA at 2015 [56]. The microbe community may vary with culture duration, unfortunately, the co-existing bacteria of *Chattonella* cultures were not monitored accordingly. The algicidal bacterium was found to be effective to the growth of *Chattonella* [57,58,59]. Even the co-existent bacteria group from *C. marina*, *Alteromonas*, *Pseudomonas*, and *Flexibacter* strains inhibited significantly on the growth of *C. marina* [60]. The role of bacteria also includes as the prey of *C. ovata* [49]; however, the obvious low growth rate of *C. ovata* may indicate the absence of predation behaviors of *C. ovata* in the present study.

The presence of lightly triggered hemolytic activity by *Chattonella* (Figure 5 and Figure 7 in the present study) and *Heterosigma* cells [20,61], suggesting that hemolytic activity could be initiated by light. Irradiance may affect toxin production directly by altering the intercellular system at the molecular level, or indirectly by changing with growth dynamics [44,62,63]. In the present study, the absence of light or iron limitation generally reduced PS activity (Figure 3 and Figure 9), ROS production (Figure 6) and hemolytic activity (Figure 5 and Figure 10) of both *Chattonella* species tested. Hemolytic activity was found to increase with light intensity in several other hemolytic toxin producers, such as *Heterosigma akashiwo* [64] and *Phaeocystis pouchetii* [65]. In contrast, an increasing HA were observed in *C. marina* in the dark when bioassays were maintained at 4 °C [44], as well as the no significant response of HA under high temperature (26 °C) and high irradiance (200 μmol photons m^−2^ s^−1^) [5]. The differenct response may possibly due to the low biomass of *Chattonella* or low capability of producing HA of the late exponential growth phase of collecting cells [44]. Noticing that the HA was displayed in the units of 50,000 cells, therefore, the HA in the present study was excluded the effect of cell biomass.

The significant difference in the relative concentration of hemolytic toxin of *Chattonella* under low light (<*I_100_*) or Fe, and high light (>*I_100_*) or iron (Fe_+_ and Fe_++_) was observed during exponential growth, but values remained relatively constant, i.e., at 80% in *C. marina* and 75% in *C. ovata* when cells reached the stationary growth stage. The lytic effect of *Chattonella* on blood cells were found in the isolates of Japan [5,20], US and Mexico [44,66] when cell aged. However, the declined HA (per 50,000 cells) under the stress of high light or iron level (Figure 5 and Figure 10) were highly likely related to the level of hemolytic compounds, and less likely to the ruptured cells. Active production of phycotoxin during the exponential growth stage was also commonly observed in the dinoflagellate *Dinophysis acuminata*, a diarrhetic shellfish poisoning (DSP) producer, followed by accumulation of DSP toxins during the stationary stage [63,67]. Similarly, the production of paralytic shellfish toxins (PST) was highest during exponential growth of *Alexandrium tamarense* [62,68]. *Karlodinium micrum* (=*Karlodinium veneficum*) showed positive hemolytic activity during both exponential and stationary growth stages [69].

Coupling between photosynthetic activity and HA indicated a direct interaction between HA and exogenous stress. The differential response of *C. marina* and *C. ovata* hemolytic activity may be attributable to differences in adaptation to light during photosynthesis in the two species (Figure 2c,d), response to iron (Figure 8c,d), ROS stress (Figure 6a,b) or variation in photopigment concentrations (Figure 4).

### 3.2. Toxinological Mechanism of Hemolytic Activity

The maximum quantum yield (F_v_/F_m_) is an essential indicator of algal cell health status. Changes in F_v_/F_m_ have been observed when algae are exposed to endogenous or exogenous stressors, such as light [52,70,71], temperature [71], salinity [72], iron and algistat addition [73,74,75,76]. A significant decline in F_v_/F_m_ of *C. marina* and *C. ovata* was shown in the present study under high light (Figure 3), in the dark (Figure 7), under iron depleted conditions (Figure 9) and the presence of PSII inhibitors (Figure 11), suggesting inactivation of PSII reaction center (RC) complexes and disruption of the electron transport chain [77,78]. In phototrophs, photon energy captured by light harvest centers is either used for photosynthesis (i.e., effective quantum yield, Yield) or for fluorescence emission or heat dissipation, i.e., non- photochemical quenching, NPQ [79]. Reduced rETR and Yield (Figure 3, Figure 7, Figure 9 and Figure 11) indicate a high level of energy dissipation and potential damage to PSII reaction centers [80]. Thus, in the present study, the decrease in PSII efficiency was associated with slow growth (Figure 2 and Figure 9) and reduced Chl *a* concentration in *C. marina* (Figure 4(d1)) under stressed conditions, reflecting disruption of normal energy pathways in the algae.

The excess energy, driven by exogenous stress, had a negative effect on the diatom *Phaeodactylum tricornutum* [81,82], dinoflagellate *Prorocentrum donghaiense* [83,84], prymnesiophyte *Phaeocystis globosa* [84,85], estuarine phytoplankton [24], and polar phytoplankton in the Polar Frontal Zone and Antarctic waters [86]. The antioxidative defense system is initiated to scavenge excess ROS [82,87]. In the present study, a large amount of ROS was produced by *C. ovata* compared to *C. marina* (Figure 6) and higher ROS production was reported in *C. antiqua* than *C. marina* [4,44,78], potentially due to regulation of the photoprotective system or xanthophyll cycle of *C. marina* (namely significantly high amount of zeaxanthin and diadinoxanthin, Figure 4e,f), and/or by dissipation of the extra energy via fluorescence or heat [36,44,78,88]. The xanthophyll cycle consists of xanthophyll, viola, antheraxanthin and zea [89,90]. Similarly, expression of xanthophyll cycle interconversion in *Chattonella* was most likely related to the production of ion superoxide (O^2−^) [52], which may participate in the *C. marina* iron- uptake process [32]. High production of *C. marina* diadinoxanthin or zea would indicate that phototrophs were under stress (Figure 4(e1,f1)), compared to *C. ovata* (Figure 4(d2,e2)), suggesting that xanthophyll pigments play a role in dissipating excess excitation energy in the PS II of *C. marina*.

Due to their high production under stress conditions, hemolytic toxins have been considered to be secondary natural products [85]. Stress would be indicated by either limited growth or photosynthetic activity [80]. Therefore, we pose the question: is hemolytic activity involved in the photoprotective system of *Chattonella*? In the present study, the relationship between hemolytic activity and ROS in all treatments (Supplementary Figure S1a,b), light (Supplementary Figure S1c,d), iron (Supplementary Figure S1e,f), and photoprotective pigments (PPPs, Supplementary Figure S2) were examined. The response of hemolytic activity vs. ROS under all treatments was positive in both species but not significant (Supplementary Figure S1a,b). The photoprotective system may function in *C. marina*, resulting in low ROS production (Figure 4a,c,e). Positive relationships were consistent in all cases, especially under iron stress (Supplementary Figure S1e). The relationship with ROS production in *C. ovata* showed a significantly lower correlation, and a negative response in the iron treatment (Supplementary Figure S1f). Therefore, the synergistic effects of ROS and ichthyotoxin production, or stimulation of toxin production by ROS was not detected in the present study (Supplementary Figure S1). This finding differs from reports for other *C. marina* isolates, indicating that ROS are synergistically involved in ichthyotoxicity through lipid peroxidation [13]. The inconsistent response of hemolytic activity to ROS in *Chattonella* is also shown by the conflicting response of hemolytic activity to PS II energy fluxes (Supplementary Figure S2) in *C. marina* and *C. ovata*. A significant, positive relationship was found in the present study between PPPs, involved in the xanthophyll cycle of *Chattonella*, and the production of hemolytic toxin (Supplementary Figure S3). The above results all suggest that hemolytic toxin compounds may be involved in energy transfer of accessory pigments, but not in the PSII photoprotective system of *Chattonella*.

We here pose a second question: is hemolytic activity involved in the electron transport chain of PSII in *Chattonella*? Photosynthetic capacity was significantly reduced by the addition of photosynthetic inhibitors (Figure 11), as reported in many other algae, e.g., atrazine was reported to inhibit the F_v_/F_m_ of *P. tricornutum* and *Chlorella* sp. [81,87], resulting in excess electron transport energy dissipation, and diuron and atrazine both reduced the Yield and rETR of *Symbiodinium* spp. [91]. As illustrated in Figure 1, diuron prevents electron transfer from QA to QB [92], while DBMIB is known to block the electron transport from PQ to Cyt b6/f [93]. Atrazine targets the QB plastoquin single-binding niche in the D1 protein of PSII, blocking electron transport from PSII [94]. In turn, paraquat diverts electrons away from the reducing side of PSI by accepting electrons from Fe-S centers and/or ferredoxin, preventing the electron transfer to NADP [94]. In the present study, the response of *C. marina* and *C. ovata* (except for that to paraquat at 7.5 mg L^−1^) indicated that the photosynthetic system was greatly affected by these inhibitors (Figure 11), resulting in significant downgrading of F_v_/F_m_, Yield and rETR within an hour of exposure.

However, the role of these herbicides is known to differ among different phototrophs. Chalifour and Juneau reported that growth and microcystin toxin production of *Microcysis aeruginosa* were inhibited by atrazine [95], whereas paraquat induced a 90% increase in microcystin toxin production [96]. *Chattonella subsalsa* was able to produce more hemolytic toxins when stressed by atrazine under low N and P conditions, but toxin production was inhibited under nutrient-replete conditions [66]. In the present study, the significance of down-regulated HA in *C. marina* (Figure 11a) and lack of response in *C. ovata* (Figure 11b) to all four photosynthetic inhibitors may result from the block of photosynthesis, but not during chain I and II electron transport (Figure 1) of *Chattonella* spp.

Finally, a third question is addressed in this study: will hemolytic activity be involved in the energy transport through the light-harvesting antenna pigments? As members of the Raphidophyceae, *Chattonella* species contain the pigments Chl *a*, *c1*, *c2*, fuco, viola, β-carotene, etc [97]. The light-harvesting complex is made up of fucoxanthin and the chl *a*/*c* complex [98]; Chl *c* compounds are unique light-harvesting pigments with a cyclic tetrapyrrol structure [99,100]. Photoautotrophic species within the Chrysophyceae [101], Raphidophyceae and Haptophyceae were reported to contain chl *c2* at amounts that vary largely due to environmental conditions [88,102,103]. The biosynthesis of chl *c* follows a multi-branched pathway and in *Chattonella* in the present study, Mg DVP, chl *c2* and chl *a* were assumed to be involved based on reports by Mysliwa-Kurdziel et al. [100]. Under this assumption, Mg DVP is the substrate in chl *c* synthesis and is converted to chl *c2* or chl *a*.

**Figure 12:**
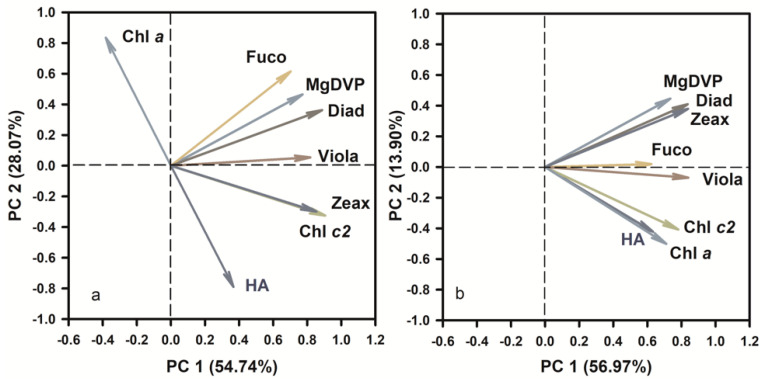
Results of principal component analysis (PCA) of hemolytic activity (HA) and the concentrations of photosynthetic pigments of *Chattonella marina* (**a**) and *C. ovata* (**b**). Chl *a*: chlorophyll *a*, Chl *c2*: chlorophyll *c2*, Fuco: fucoxanthin, Viola: violaxanthin, Diad: diadinoxathin, Zea: zeaxanthin.

**Figure 13:**
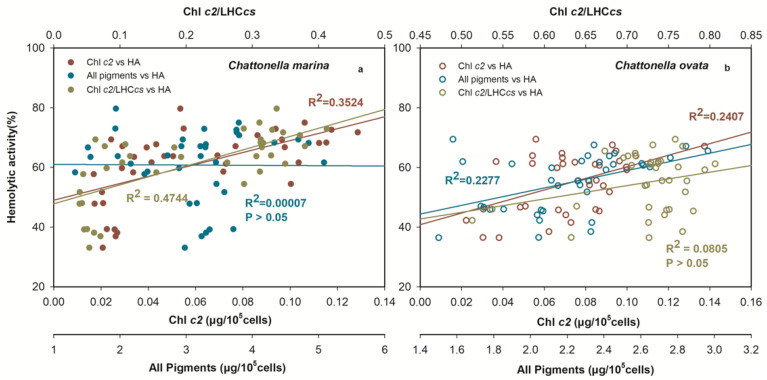
Linear relationship between hemolytic activity and all photosynthetic pigments, ratio of chlorophyll *c2* to chl *c* biosynthetic pigments and chl *c2* of *Chattonella marina* (**a**) and *C. ovata* (**b**). *R*^2^ = coefficient of determination of the fitted linear regressions.

Principal Component Analysis (PCA) was conducted in this study to determine the principal components of photosynthetic pigments in hemolytic activity (Figure 12). The scores of the first two principal components (PC1 and PC2) reached 54.7 and 28.1% (Figure 12a), 57.0 and 13.9% (Figure 12b) for *C. marina* and *C. ovata*, respectively. The HA was apparent in highly positive PC1 space of both *Chattonella* and appeared quite separated from pigments of Fuco, MgDVP and Diad. However, HA were found in a positive relationship to Chl *c2*, but negative to Chl *a* in *C. marina* (Figure 12a), whereas, both Chl *a* and *c2* were positively related to HA in *C. ovata* (Figure 12b). Chlorophyll *c2* and chl *a* were the top-ranking pigments and thus most likely to be related to the production of hemolytic toxin. Therefore, further statistical analysis was conducted to determine the relationship between hemolytic toxin activity and all pigments, the ratio of chl *c2* to the light-harvesting antenna with chlorophylls *c* (LHC*cs*), and with chl *c2* (Figure 13) of exponentially growing *C. marina* and *C. ovata* (days 5, 7 and 9). Hemolytic activity showed a significant positive correlation with Chl *c2* in both species (R^2^ = 0.35 and 0.24 for *C. marina* and *C. ovata* respectively (Figure 13a,b), whereas the relationship to all pigments or ratio of chl *c2* to LHC*cs* was not significant. Chlorophyll *c2* was not a dominant pigment of *Chattonella*, as it only made up ~2% of the accessory pool of light-harvesting pigments. However, this low amount of chl *c* was reported to have a potentially toxic effect [27,104]. In *Sargassum horneri* chl *c2* suppressed the degranulation of rat basophilic leukaemia cells [104]. Additionally, an analogue of chl *c*, extracted from the marine brown alga *Eisenia bicyclis* blocked the activity of a fish rhabdovirus [27]. However, not all chl *c2* containing algae have been reported to be toxic. Therefore, it is possible that these pigment analogues (non-hemolytic or per-hemolytic or low-potency hemolytic toxins), acted as electron transporters by accepting electrons and were converted into unstable hemolytic toxins. Further evidence at the molecular level is still needed to resolve this. However, our current results contribute a novel potential interpretation of the mechanism of hemolytic activity.

## 4. Materials and Methods

### 4.1. Algae and Culture Conditions

*Chattonella marina* (CMHK) and *C. ovata* (COHK), previously isolated from Hong Kong waters, South China Sea, at 2002 and 2003, respectively, were provided by the Research Center of Harmful Algae and Marine Biology, Jinan University. Stock non-axenic cultures were maintained at 20 °C, 28 salinity and 100 µmol m^−2^ s^−1^ of light intensity with a 12:12 light:dark cycle.

Growth rate (*µ*) of *C. marina* or *C. ovata* was calculated using the following equation:

(1) $$\mu =\frac{\ln(C_{2}/C_{1})}{t_{2}-t_{1}}$$

where *C*_2_ and *C*_1_ are the cell numbers at the end of the logarithmic phase (*t*_2_) and at time zero (*t*_1_), respectively [105].

### 4.2. Effects of Light and Iron (Experiment I)

Five different light intensities, 30, 60, 100, 180 and 270 µmol m^−2^ s^−^^1^, and three different FeCl_3_ concentrations: 0, 0.12, and 11.6 µmol L^−1^, were tested separately for *C. marina* and *C. ovata* in Experiment I. *Chattonella marina* and *C. ovata* were each incubated in artificial seawater with f/2-Si medium and preconditioned for two generations [106]. All treatments were conducted in triplicate. Samples for hemolytic activity, ROS, cell density and photosynthetic fluorescence parameters were collected every 2 or 3 days.

### 4.3. Daily Light:Dark Cycle Variation (Experiment II)

*Chattonella marina* or *C. ovata* cells in exponential growth stage were inoculated in triplicate in f/2-Si medium under a light intensity of 100 µmol m^−2^ s^−1^ and 12:12 light:dark cycle (started at 9 a.m.), salinity of 28 and temperature of 24 °C, one hour before the light cycle started. Samples for ROS, toxin concentrations and photosystem parameters were collected every 4 h over a daily cycle (24 h).

### 4.4. Effects of Photosynthetic Electron Transport Inhibitors (Experiment III)

Four photosynthetic inhibitors, diuron [3-(3,4-dichlorophenyl)-1,1-dimethylurea], atrazine, dibromothymoquinone (DBMIB) and paraquat (N, N′-dimethyl-4,4′-bipyridinium dichloride), were used in Experiment III. Acetone-dissolved diuron, atrazine and DBMIB and water-dissolved paraquat were added to *C. marina* or *C. ovata* 5-day cultures in exponential growth stage a final concentration of 0.075, 0.05, 0.05 and 7.5 mg L^−1^, respectively. A negative control was established by culturing algae with the inhibitors in their original solvents. i.e., acetone for diuron, atrazine for DBMIB and distilled water for paraquat. All cultures were run in triplicate and grown at 24 °C, salinity of 28 and 100 µmol m^−2^ s^−1^ light intensity. Hemolytic activity, F_v_/F_m_, (quantum yield) and rETR were measured after 1 h-incubation; ROS were measured under diuron and DBMIB exposure conditions.

### 4.5. Data Analysis

#### 4.5.1. Hemolysis Assay

The hemolytic activity of *C. marina* and *C. ovata* was quantified using rabbit blood erythrocytes following Eschbach et al. and Ling and Trick [61,107]. Erythrocytes were directly obtained from the rabbit’s ear (New Zealand White rabbit), washed twice with phosphate- buffered saline (PBS) and stored at 4 °C for up to 7 days. For hemolysis analysis, the erythrocytes were washed again and diluted to a final concentration of 5% (*v*/*v*) in PBS. The previously prepared *C*. *marina* or *C. ovata* suspension of prepared erythrocytes (150 µL) was mixed into a 1 mL centrifuge tube, and set as test samples (*A_e_*). The same amount of algal suspension, incubated in PBS, served as control (*A_a_*) to account for algal background absorbance. The complete lysis of erythrocytes (exposed to 2% digitonin) served as positive control (*A_p_*) and the prepared erythrocytes were the negative control (*A_n_*). All samples were incubated for 5 h at 25 °C under a light intensity of 100 µmol m^−2^ s^−1^. Then, the samples were centrifuged at 3000 rpm, 25 °C for 10 min. A volume of 200 µL of the supernatant from each tube was transferred to a 96-well microplate (Corning, Glendale, AZ, USA) and the released hemoglobin absorbance was measured at 414 nm in a Microplate Reader (Biotek Synergy HT, Winooski, VT, USA).

Hemolytic activity was expressed as a percentage (%) according to Ling and Trick [61]:

(2) $$\%\;\mathrm{hemolytic}\;\mathrm{activity}=\frac{A_{e}-A_{a}-A_{n}}{A_{p}}\times 100\%$$

where *A_e_, A_a_, A_n_* and *A_p_* are the absorption at 414 nm of the sample incubated with algae + erythrocytes (test samples), algae only (background), healthy erythrocytes (negative control), and lysed erythrocytes (positive control), respectively.

The hemolytic 50% effective concentration of *C*. *marina* and *C. ovata*, EC_50_, was first established by dose-effect simulation. Concentrations of 3 × 10^3^, 7.5 × 10^3^, 1.5 × 10^4^, 3 × 10^4^, 6 × 10^4^, 1 × 10^5^, and 2 × 10^5^ cells mL^−1^ were used. A final EC_50_ value of 5 × 10^4^ cells mL^−1^ for *C. marina* or *C. ovata* was obtained. Therefore, all toxin samples were prepared to yield a final test concentration of 5 × 10^4^ cell mL^−1^. Thus, ~10 to 20 mL of *C. marina* or *C. ovata* from each treatment were centrifuged at 3000 rpm at 4 °C for 10 min. Pellets were resuspended in assay buffer [107] to yield 5 × 10^4^ cells mL^−1^, and the suspension was ultrasonicated (Sonifier 540, Branson, Brookfield, CT, USA) on ice at 10% cycle (650 W) for 50 s (2 s pulse on, 1 s pulse off), to be ready for the hemolysis assay. Toxin production rate was calculated over the entire growth cycle of *C. marina* or *C. ovata*, by dividing the percent difference by the number samplings days, expressed in units of% hemolytic activity of 5 × 10^4^ cells mL^−1^ per day.

#### 4.5.2. Hydrogen Peroxide (H_2_O_2_) Assay

Hydrogen peroxide (H_2_O_2_), of all the reactive oxygen species (ROS), was recognized as the most stable compound in seawater [108], therefore, was selected as an indicator of ROS. Hydrogen peroxide was determined using the H_2_O_2_ pHPA assay [109,110]. Briefly, horseradish peroxidase (HRP) reacts with H_2_O_2_ in the samples and then oxidizes the compound para-hydroxyphenylacetic acid (pHPA), resulting in the formation of the fluorescent pHPA dimer, which was recorded by a UV spectrophotometer (Shimadzu, Kyoto, Japan) with excitation at 320 nm and emission at 405 nm for readout of the amount of H_2_O_2_. Standard H_2_O_2_ stock solutions were prepared at concentrations of 20, 40, 60, 80, 100, 120 and 140 µmol L^−1^. Two mL of standard H_2_O_2_ stock solution or sample were first added to 1mL of 1.5 mmol L^−1^ pHPA and 30 µL of 10 mg mL^−1^ HRP (horseradish peroxidase, Aladdin, China). The absorbance difference before and after adding 30 µL of 10 mg mL^−1^ catalase (CAT, Aladdin, China) was recorded and used to determine the concentration of H_2_O_2_. 

#### 4.5.3. Measurement of Photosynthetic Fluorescence

Photosynthetic fluorescence parameters were measured using a pulse amplitude modulation fluorometer (Phyto-PAM, Walz, Effeltrich, Germany). Samples were pre-adapted in the dark for 5 min at the recording temperature. The maximum quantum yield of PSII (F_v_/F_m_), the effective PSII quantum yield (Yield) and the relative electron transfer (rETR) were obtained in Report windows of the Phyto-PAM (Walz, Effeltrich, Germany). 

#### 4.5.4. Photopigment Analysis

Culture samples (15 mL) were filtered through Whatman GF/F glass fiber filters (0.7 µm nominal pore size, 25 mm diameter), which were stored in 95% methanol in darkness at −80 °C. Pigment concentrations were determined using an Agilent 1200 HPLC system (Agilent, Santa Clara, CA, USA) with a C8 column (Waters) (4.6 × 150 mm, 3.5 μm) following methods of Zapata et al. [111]. Pigment standards of chlorophyll *c3*/*c2*/*b*/*a*, Mg-2,4-divinylpheoporphyrin (Mg DVP), peridinin (perid), pheophorbide *a*, 19-but-fucoxanthin (but-fuco), fucoxanthin (fuco), neoxanthin (neo), prasinoxanthin (pras), violaxanthin (viola), 19-Hex-fucoxanthin (hex-fuco), diadinoxanthin (diadino), alloxanthin (allo), myxoxanthophyll, diatoxanthin, zeaxanthin (zea), canthaxanthin, β-cryptoxanthin, pheophytin *a* and carotene were purchased from DHI Inc. (Aarhus, Denmark).

Subsamples for pigment analysis were collected only during the exponential growth phase of the two *Chattonella* species, at Day 5, 7 and 9, therefore, were classified as early, middle and late exponential growth phase, respectively.

### 4.6. Statistical Analysis

All statistical analysis was conducted using SigmaPlot v. 14.0 software. The correlations of F_v_/F_m_, Yield, rETR and specific pigments with hemolytic activity were analyzed by linear regression. One-way repeated measures ANOVA with Holm-Sidak pairwise comparisons were used to test for the effects of light intensity, temperature or iron on the growth rate of *C. marina* or *C. ovata*, those of light/dark cycle on hemolytic activity, light on pigment content, and photosynthetic electron block on hemolytic activity, F_v_/F_m_, Yield or rETR. Two-way repeated measures ANOVA was used in the time series experiment, i.e., light, temperature and iron effects on growth, hemolytic activity, F_v_/F_m_, Yield or rETR; *p* was set at 0.05. Principal component analysis (PCA) was performed on the value of HA and all seven detected pigments (n = 36) of the two *Chattonella* species, to help understand the linear relationship between HA and pigmentation.

## 5. Conclusions

This study focuses on the toxinological mechanism/s of hemolytic activity during photosynthesis of two *Chattonella* spp., *C. marina* and *C. ovata*, with the processes of PSII photosynthetic efficiency, photoprotective regulation, and light-harvesting antenna pigments. Hemolytic activity of both species was light-dependent, increasing at low light intensity (*I_30_*~*I_100_*), and was generated during cell division, i.e., during exponential growth of *C. ovata* under all light conditions tested, and that of *C. marina* at low light (*I_30_*~*I_60_*). Healthy, more actively photosynthetic cells of *C. marina* produced more hemolytic toxin, in contrast to *C. ovata* that was capable of producing high amounts of hemolytic toxin only under stress. Hemolytic activity in the two *Chattonella* species did not appear to be associated with the photoprotective system, i.e., xanthophyll cycle and ROS regulation, or to be generated during the photosynthetic electron transport chain in *Chattonella*. However, hemolytic activity was closely related to the concentration of light-harvesting antenna pigments, especially chl *c2* and chl *a*, indicating that hemolytic toxin in *Chattonella* may be generated during electron/energy transfer via chl *c2* biosynthesis. However, many algae contain chl *c2* but not all have an ichthyotoxic effect. Further confirmatory studies are required, but results of this study provide a basis for future studies.

## Data Availability

All data are contained within this article and supplementary materials.

## Supplementary Materials

The following are available online at https://www.mdpi.com/article/10.3390/md19060336/s1, Figure S1: Linear relationship between percent hemolytic activity and the reactive oxygen species (ROS) production of *Chattonella marina* (a,c,e) and *C. ovata* (b,d,f) under all treatments (a,b), light only (c,d) and iron only treatment (e,f). *R*^2^ = coefficient of determination of the fitted lines., Figure S2: Linear relationship between hemolytic activity and ratio of photoprotective pigments to total pigments of *Chattonella marina* (a) and *C. ovata* (b). *R*^2^ = coefficient of determination of the fitted lines., Figure S3: Linear relationship between hemolytic activity and F_v_/F_m_ (blue), Yield (red), rETR (yellow) of *Chattonella marina* (a) and *C. ovata* (b) in varied light intensities, iron, light/dark cycle and photosynthetic blockers treatment. R^2^ = coefficient of determination of the fitted lines.
