# Supplementary material for: Hemolytic Activity in Relation to the Photosynthetic System in Chattonella marina and Chattonella ovata
Source: Mar Drugs. 2021 Jun 12;19(6):336. doi: 10.3390/md19060336 (PMC8231601; doi:10.3390/md19060336)

Supplementary Material

Hemolytic Activity in relation to the Photosynthetic System in *Chattonella marina* and *Chattonella ovata*

Ni Wu[1,2], Mengmeng Tong[3*], Siyu Gou[1], Weiji Zeng[1], Zhuoyun Xu[3], Tianjiu Jiang[1*]

[1] Research Center of Hydrobiology, Key Laboratory of Aquatic Eutrophication and Control of Harmful Algal Blooms of Guangdong Higher Education Institute, Jinan University, Guangzhou 510632, China

[2]South China Sea Institute of Planning and Environmental Research, State Oceanic Administration, Guangzhou 510300, China

[3] Ocean College, Zhejiang University, Zhoushan, 316021, China

*Corresponding author: mengmengtong@zju.edu.cn, tjiang@jnu.edu.cn

Supplementary Figure S1

Linear relationship between percent hemolytic activity and the reactive oxygen species (ROS) production of *Chattonella marina* (a,c,e) and *C. ovata* (b,d,f) under all treatments (a,b), light only (c,d) and iron only treatment (e,f). R^2 = coefficient of determination of the fitted lines.

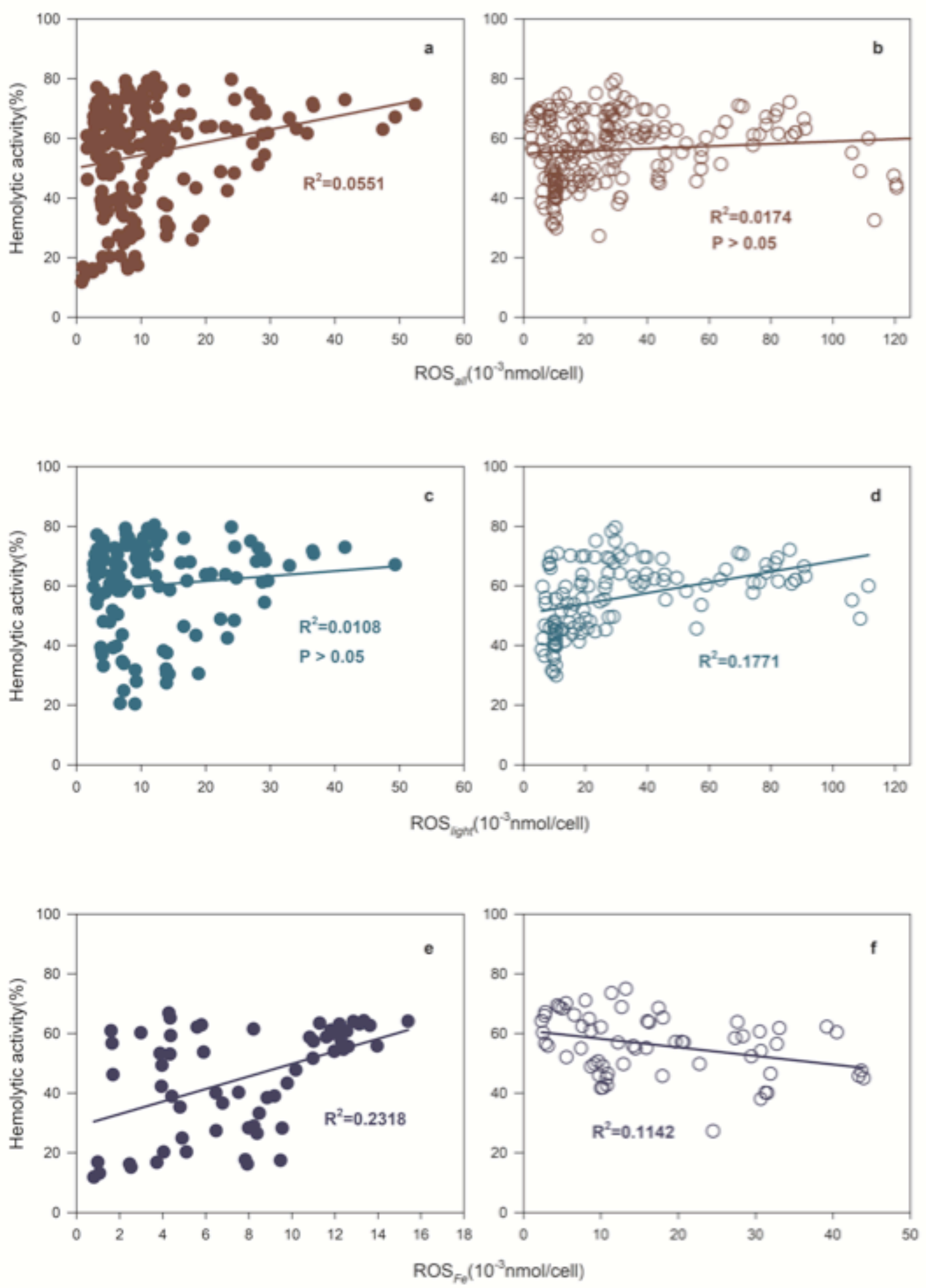

Supplementary Figure S2

Linear relationship between hemolytic activity and ratio of photoprotective pigments to total pigments of *Chattonella marina* (a) and *C. ovata* (b). R^2 = coefficient of determination of the fitted lines.

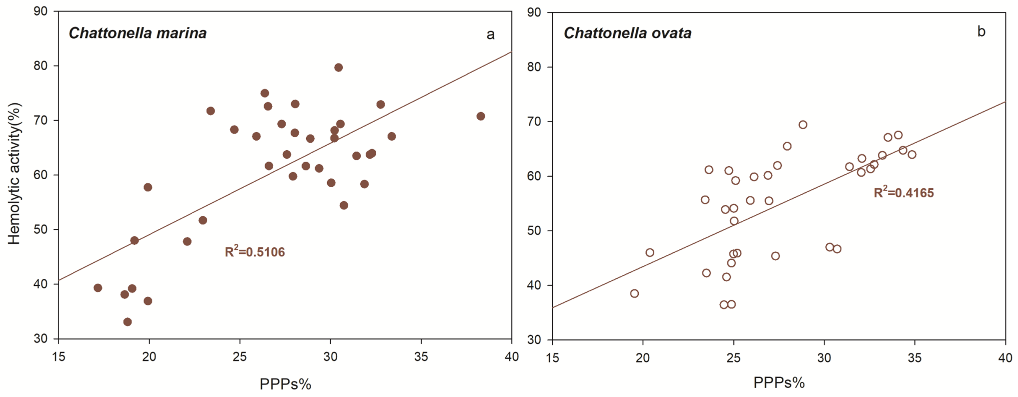

Supplementary Figure S3

Linear relationship between hemolytic activity and Fv/Fm (blue), Yield (red), rETR (yellow) of *Chattonella marina* (a) and *C. ovata* (b) in varied light intensities, iron, light/dark cycle and photosynthetic blockers treatment. R^2 = coefficient of determination of the fitted lines.

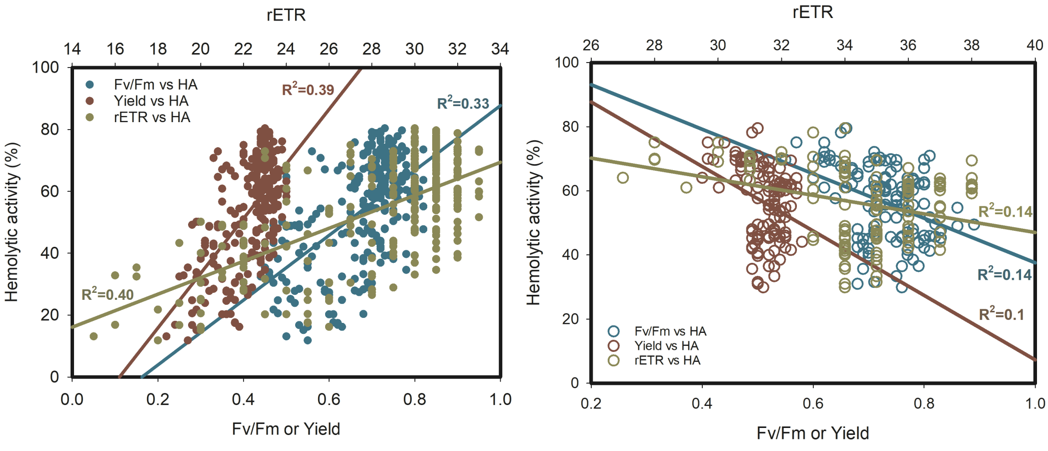

Supplement: Supplementary file 1 [file marinedrugs-19-00336-s001.zip › marinedrugs-1224680-supplementary.pdf]
